# Toward Safe Pharmacotherapy: The Interplay between Meropenem and Parenteral Nutrition Admixtures

**DOI:** 10.3390/antibiotics10020217

**Published:** 2021-02-22

**Authors:** Aleksandra Gostyńska, Ludwika Piwowarczyk, Malwina Nadolna, Anna Jelińska, Katarzyna Dettlaff, Magdalena Ogrodowczyk, Maria Popielarz-Brzezińska, Maciej Stawny

**Affiliations:** Department and Chair of Pharmaceutical Chemistry, Poznan University of Medical Sciences, 6 Grunwaldzka, 60-780 Poznań, Poland; gostynska.aleksandra@spsk2.pl (A.G.); ludwika.piwowarczyk@wp.eu (L.P.); malwina_nadolna125@interia.pl (M.N.); ajelinsk@ump.edu.pl (A.J.); dettlaff@ump.edu.pl (K.D.); mogrodo@ump.edu.pl (M.O.); mpopiel@ump.edu.pl (M.P.-B.)

**Keywords:** safe pharmacotherapy, meropenem, Y-site administration, parenteral nutrition, drug compatibility

## Abstract

Simultaneous administration of parenteral nutrition (PN) admixtures with intravenous antibiotics is a common clinical problem. Coadministration of drugs incompatible with PN admixture may affect its stability, especially in the context of lipid droplet size, which is a crucial parameter for patient safety. In the present study, we investigate the in vitro compatibility of meropenem (Meropenem 1000, MPM) with five commercial PN admixtures used worldwide: Kabiven, Olimel N9E, Nutriflex Lipid Special, Nutriflex Omega Special, and SmofKabiven. The appropriate volumetric ratios, reflecting their clinical practice ratios, were used to prepare the MPM–PN admixture samples. Physicochemical properties of MPM–PN admixtures samples were determined upon preparation and after four hours of storage. The pH changes for all MPM–PN admixtures samples did not exceed the assumed level of acceptability and ranged from 6.41 to 7.42. After four hours of storage, the osmolarity changes were ±3%, except MPM–Olimel N9E samples, for which differences from 7% to 11% were observed. The adopted level of acceptability of changes in zeta potential after four hours of storage (±3 mV) was met for MPM–Kabiven, MPM–Nutriflex Lipid Special, and MPM–Nutriflex Omega Special. The mean droplet diameter for all samples was below 500 nm. However, only in the case of Nutriflex Lipid Special and Nutriflex Omega Special, the addition of MPM did not cause the formation of the second fraction of lipid droplets. The coadministration of MPM via Y-site with Kabiven, Olimel N9E, and Smofkabiven should be avoided due to osmolarity fluctuations (MPM–Olimel), significant differences in zeta potential (MPM–Olimel, MPM–Smofkabiven), and the presence of the second fraction of lipid droplets >1000 nm (MPM–Kabiven, MPM–Olimel, and MPM–Smofkabiven). The assumed acceptance criteria for MPM compatibility of MPM with PN admixtures were met only for Nutriflex Lipid Special and Nutriflex Omega Special in 1:1, 2:1, and 4:1 volume ratios.

## 1. Introduction

The simultaneous administration of drugs is a frequent clinical practice. This procedure applies to patients with a limited number of vascular accesses or patients in whom the insertion of subsequent vascular accesses is impossible or inadvisable [1]. Such a situation is common in intensive care units where patients need complex pharmacotherapy, including parenteral nutrition and antibiotics administration. Meropenem (MPM) is an antibiotic reserved for the treatment of severe infections. It belongs to the beta-lactams, carbapenem group, and it acts by inhibiting the synthesis of the bacterial cell wall by attaching to penicillin-binding protein (PBP). This antibiotic is resistant to the action of beta-lactamase and renal dehydropeptidase I. MPM, after intravenous administration, binds to plasma proteins in 2% and distributes to the lungs, bile, skin, muscles, the peritoneal cavity, and the cerebrospinal fluid. Due to the wide range of antibacterial effect on both gram-positive (*Streptococcus agalactiae*, *Enterococcus faecalis*, *Staphylococcus aureus*, *Streptococcus pneumoniae*, *Clostridium perfringens*) and gram-negative (*Escherichia coli*, *Haemophilus influenzae*, *Klebsiella pneumoniae*, *Serratia pneumoniae*) bacteria, it is commonly used to treat different serious infections in critically ill patients.

The use of the Y-site for the simultaneous administration of intravenous drugs and parenteral nutrition (PN) is not recommended if there is a lack of data supporting its safety. Such an administration may endanger patients’ health and life due to the possibility of interaction between the drug and the PN admixture. It was previously shown that the absence of data confirming the compatibility of two coadministered drugs via the intravenous route leads to high risk of improper administration [2,3]. The most frequent interactions involved drug or PN admixture ingredient precipitation, the formation of large lipid droplets exceeding the critical acceptance limit for intravenous administration, loss of homogeneity of the oil-in-water system, or color change. Such incompatibilities were reported in previous studies [4,5,6,7,8,9,10,11,12,13,14,15] (Table 1). Interpretation of the results of drug–PN compatibility tests and their adaptation into clinical practice should be based not only on the conclusion whether the drug is or is not compatible with PN admixture, but also involve a detailed analysis of the pharmaceutical preparation of the drug (pH, excipients, solubilizers), the composition of the PN admixture (electrolyte content, type of lipid emulsion), and its physicochemical properties (pH, osmolarity) [2,16,17,18].

The concept of PN, developed in the 1960s by Professor Stanley Dudrick, assumes the simultaneous supply of all nutrients from one container. PN therapy can be implemented in two ways, namely, compounded PN admixtures or ready-to-use (RTU) PN admixture produced by the pharmaceutical industry. The first solution, i.e., a compounded PN admixture, is most often used in relation to long-term parenterally fed patients who require adjusting their composition to clinical conditions and obligatorily in pediatric and neonatal patients. The remaining patients who require PN admixture administration receive the most commonly RTU PN mixtures produced in a wide range of compositions and volumes. They can meet the energy and nutritional needs of most patients. Compatibility studies concerning RTU, commercially produced PN admixtures are versatile and clinically useful due to the worldwide availability of such preparations and their more frequent use than compounded PN admixtures.

In the present study, we investigate the in vitro compatibility of meropenem (Meropenem 1000, MPM) with five commercial PN admixtures used worldwide: Kabiven 1540 mL (Kabiven), SmofKabiven 1477 mL (Smofkabiven), Olimel N9E 1500 mL (Olimel), Nutriflex Omega Special 1875 mL (Nutriflex OS), Nutriflex Lipid Special 1875 mL (Nutriflex LS). The methodology of compatibility studies is not established. According to some researchers’ opinions, in drug–PN admixture compatibility tests, it is necessary to determine the physicochemical compatibility (interplay between mixed drugs). The stability of the drug (changes in the drug concentration) can be omitted, as it is assumed to be less critical due to the short contact time of the drug with PN admixture during the Y-site administration [10,11]. Following such recommendation, we performed the compatibility studies analyzing changes in pH, osmolarity, mean droplet diameter (MDD) of lipid emulsion, and zeta potential.

## 2. Results

The addition of MPM to PN admixtures did not cause any color changes, signs of lipid emulsion destabilization, or precipitate formation, which two independent observers confirmed. The pH of PN admixtures did not change significantly after the addition of MPM. For all tested MPM–PN admixture samples, a decrease in pH was observed, which further decreased after four hours of storage. The maximum pH reduction at t = 4 h was observed for MPM–Olimel 1:1 (change by 0.13), while for the remaining samples, the pH change was not greater than 0.07. A concentration-dependent decrease in osmolarity was observed, which significantly differed from PN admixtures’ osmolarity without the addition of MPM. After four hours of storage, the observed osmolarity changes did not have a directional character and did not exceed ±3%, except for MPM–Olimel samples. In this case, osmolality differences depended on the drug–PN admixtures volume ratio and ranged from 7% to 11%.

The addition of MPM to PN admixtures reduced the zeta potential, but no linear correlation between MPM concentration and this parameter was found. The physical characteristics of MPM–PN admixture samples are presented in Table 2.

Only in the case of MPM–Olimel (4:1 volume ratio) was the presence of two fractions of lipid emulsion particles observed immediately after sample preparation (t = 0 h): dF1 = 282 ± 6 and dF2 = 5170 ± 9. At t = 4 h, the second fraction of lipid emulsion particles was observed for MPM–Olimel samples in 1:1 and 2:1 volume ratios, for MPM–Kabiven, and MPM–Smofkabiven in volume ratios 2:1 and 4:1. The highest percentage of the second fraction of particles (3.1%) occurred for MPM–Olimel in the 4:1 volume ratio. Simultaneously, a proportional increase in the percentage of dF2 to the MPM concentration in the sample was observed. Interestingly, for all samples, including those with dF2, the MDD was not greater than 500 nm. The dF1 ranged from 220 nm to 330 nm. The smallest MDD value at t = 0 h was recorded for MPM–Nutriflex LS and MPM–Nutriflex OS samples (from 209 nm ± 2 nm to 212 nm ± 2 nm and from 215 nm ± 1 nm to 224 nm ± 2 nm, respectively), and the largest for MPM–Kabiven (from 276 nm ± 5 nm to 280 nm ± 3 nm). The four hours storage of MPM–PN admixtures samples did not cause significant changes in MDD, except the MPM–Olimel samples, for which MDD at t = 4 h differed significantly (*p* < 0.05) from MDD obtained immediately after receiving the samples. For all MPM–PN admixture samples, the observed polydispersity index (PDI) at t = 0 h was <0.07 and did not change during storage, except for samples for which the presence of dF2 was observed (PDI increased up to 0.21). The MDD, dF1, dF2, and PDI values are presented in Table 3.

## 3. Discussion

So far, the MPM and PN admixtures’ compatibility tests were undertaken by two groups of researchers [4,19]. Trissel et al. [19] showed that MPM is compatible with the nine compounded PN admixtures. According to their research methodology, the compatibility was proven by lack of visible particles, lack of oiling (formation of a layer of free oil on the top of a disrupted lipid emulsion layer), cracking, creaming, color change, or gas evolution. Bouchoud et al. [4] determined MPM compatibility at a concentration of 50 mg/mL (bolus administration) with Nutriflex LS. As acceptance criteria, the researchers adopted: change in pH ≤ 0.2, no lipid droplets > 15 μm visible in five microscopic images, not more than 90 lipid droplets from 2–15 μm in size in five microscopic images, changes of MDD during storage < 10%, and PFAT5 < 0.04% (PFAT5: the percentage of fat residing in globules larger than 5 μm). MPM–Nutriflex LS in a 1:1 volume ratio met these criteria and was found to be compatible.

Our research used MPM solution in a concentration of 8.33 mg/mL (1000 mg MPM dissolved in 120 mL of 0.9% sodium chloride), which was administrated in clinical practice in 30–60 min infusion. We mixed each of the PN admixtures with MPM in three-volume ratios (1:1, 2:1, and 4:1), which simulated the coadministration of those drugs via Y-site. The mixing ratios were calculated based on both preparations’ infusion rates, i.e., the drug and the PN admixture. It should be emphasized that the time of infusion of MPM and PN admixture lasts 15–30 min and 16 to 24 h, respectively. The time of coexisting of both drugs in the infusion line can be counted in minutes. The exact time of contact can be calculated on the basis of the infusion rates of co-infused drugs, the volume of the infusion line, and the placement of Y-site in the infusion line. In this study, two endpoints were applied, namely, immediately after preparation and after 4 h, in order to fully characterize the potential incompatibilities of the studied drugs during infusion. The second endpoint (four hours) was chosen to characterize possible interactions in case of any infusion time variation or the volume of the infusion line.

The following criteria were set to consider the MPM–PN admixture samples as compatible: pH change not greater than ±0.2 (after 4 h of storage), osmolarity change not greater than ±5% (after 4 h of storage), difference in zeta potential not greater than ±3 mV (after 4 h of storage), lack of the second fraction of lipid particles >1000 nm, and following United States Pharmacopoeia (USP), the MDD of lipid emulsion <500 nm [20].

The pH changes for all MPM–PN admixture samples did not exceed the assumed level of acceptability. The amino acid solution used to prepare PN admixtures had a high buffering capacity, further increased by acetates. The addition of drugs that are weak acids or bases to PN admixtures may cause precipitation of the drug due to a change in pH and the transition of the drug from the ionized form to the nonionized one. Such a phenomenon was observed, for example, for ondansetron, whose solubility decreases at pH > 5.7 [21]. The tested MPM solution was characterized by pH = 8.03, and adjusting to pH 5.0 did not cause precipitation or opalescence of the solution. Similarly, no signs of precipitation were observed after mixing MPM with PN admixtures, with pH ranging from 5.44 to 6.26.

Osmolarity changes after four hours of storage of MPM–PN admixture samples were ±3%, except MPM–Olimel samples, for which differences from 7% to 11% were observed. Similar changes in osmolarity were observed for vancomycin–Olimel samples [7], suggesting that osmotically active ingredients are involved in drug–Olimel interactions or that metal ions are involved in the formation of lipid emulsion–drug complexes. The osmolarity of PN admixtures was >1000 mOsm/kg. Thus, the coadministration of compatible preparations should be provided into the central veins. Administration of hyperosmolar drug into peripheral veins leads to dehydration and contraction of blood cells and damage of blood vessels (phlebitis) [22].

The determination of the zeta potential, which is the potential difference between the dispersion medium and the stationary layer of fluid attached to the particle, allows evaluation of the strength of electrostatic interactions between particles in a PN admixture. The zeta potential depends on the electrolyte concentration and the pH of the sample. It was shown that lipid emulsions used in PN, due to stabilizing phospholipids, are characterized by a zeta potential of −40 to −50 mV, thus exhibiting substantial stability. Depending on the TPN admixtures’ composition, the zeta potential may even have values close to zero, in a range of −4.1 to −1.7 mV [5,6,7].

The zeta potential of MPM–PN samples at t = 0 h depended on the type of lipid emulsion, PN admixture composition, and MPM concentration. Zeta potential of MPM–PN samples ranged from −14.20 mV (MPM–Nutriflex OS in 4:1 ratio) to −34.90 mV (MPM– Nutriflex LS in 4:1 ratio). The adopted level of acceptability of changes in zeta potential after four hours of storage (±3 mV) was met for MPM–Kabiven, MPM–Nutriflex LS, and MPM–Nutriflex OS. For the remaining MPM–PN admixture samples (MPM–Olimel and MPM–Smofkabiven), the differences were above the acceptable limit and ranged from 3.00 mV to 11.70 mV. These observations suggested the dynamic impact of MPM on the strength of electrostatic interactions between lipid droplets in a PN admixture.

Lipid emulsions used in parenteral nutrition are oil-in-water (*o*/*w*) system characterizing by a particle size between 200 and 400 nm and exhibit thermodynamic instability. Factors such as temperature above 25 °C, high concentration of cations (critical aggregates concentration, CAN > 600), and exposure to oxygen may affect the stability of the *o*/*w* emulsion. The first stage of destabilization of such product is creaming, manifested by thickening of the upper layer due to the formation of larger aggregates with or without increasing the mean particle size. The next stage of destabilization of the lipid emulsion is coalescence, i.e., the release of oil drops resulting from exceeding the critical values, maintaining electrostatic differences between the inner and outer micelle layers. This step is irreversible and cracks the emulsion. The destabilization processes of the lipid emulsion may also be accompanied by a change in color, usually yellowing, due to oxidation of free fatty acids and the formation of reactive peroxides. These types of reactions are radical and initiate drug decomposition. An intravenous administration of such preparations may cause the intensification of inflammation due to intravenous administration of free radicals. Both the destabilization process of the lipid emulsion and reactive oxygen species formation may affect the lipid emulsion particles’ size. In the event of agglomerate formation, it may directly threaten patients and lead to capillary embolization.

**Figure 1:**
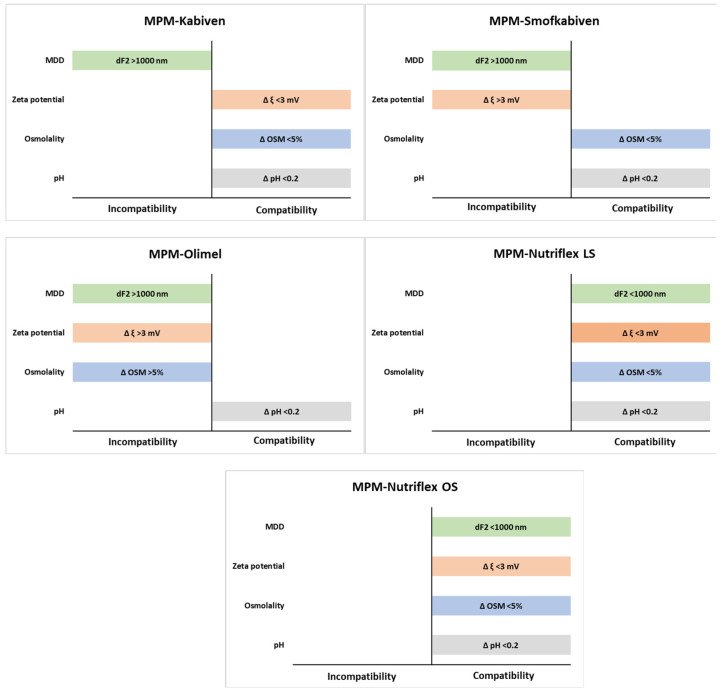
Summary of compatibility studies of MPM and parenteral nutrition admixtures.

Lipid emulsion droplet size is one of the most critical parameters that should be considered when determining PN admixture administration safety. US Pharmacopoeia [20] gives two methods for determining the size of lipid emulsion droplets, namely, the method using the dynamic light scattering (DLS) technique, for which MDD should not be greater than 500 nm, and a method based on light obscuration, for which large-diameter droplets (>5 μm) of lipid emulsions should not exceed 0.05%. Our research used the first method to determine MDD and adopted an acceptable value of 500 nm, following USP recommendations. Additionally, we introduced a second acceptance parameter, i.e., no second fraction of particle >1000 nm. The second fraction of lipid particles of larger size (~5 µm) in parenteral nutrition poses a risk to patients, as pulmonary embolism may occur. Animal studies showed that fat droplets size about 5 µm cause harm to the lungs and liver [21]. MDD was below 500 nm; however, only two of the tested PN admixtures (Nutriflex LS and Nutriflex OS) did not form the second fraction of lipid emulsion particles after MPM addition. In the case of MPM–Olimel samples in a 4:1 volume ratio, the second fraction of particles was observed immediately after sample preparation and after four hours of storage, indicating that MPM affects lipid emulsion. At t = 4 h, the second fraction of lipid emulsion particles was also observed for MPM–Olimel 1:1 (*v*/*v*) and 2:1 (*v*/*v*) and for all samples of MPM–Kabiven and MPM–Smofkabiven. The reason for the formation of larger droplets of lipid emulsion is most likely the type of lipid emulsion (fatty acids composition) and the number of emulsifiers used. The size of the large lipid droplets formed and their content depends on the sample’s MPM concentration. For all MPM–PN admixture samples, for which the presence of the second fraction of particles >1000 nm was observed, the PDIs were >0.1, indicating a decrease in the homogeneity of the oil-in-water system. The summary of conducted investigations is presented in Figure 1.

## 4. Materials and Methods

### 4.1. Sample Preparation

MPM solution (Meropenem 1000, Pfizer Europe) was prepared by adding 20.0 mL of water for injection to the vial containing 1000 mg of MPM. A total of 4.17 mL of solution was withdrawn and transferred to 25 mL of 0.9% sodium chloride solution. The MPM concentration was 8.34 mg/mL.

Five ready-to-use PN admixtures were used in this study: Kabiven 1540 mL (Kabiven), Fresenius Kabi AB, Uppsala, Sweden; SmofKabiven 1477 mL (Smofkabiven), Fresenius Kabi AB, Sweden; Olimel N9E 1500 mL (Olimel) Baxter, Poland; Nutriflex Omega Special 1875 mL (Nutriflex OS) B. Braun Melsungen AG, Melsungen, Germany; and Nutriflex Lipid Special 1875 mL (Nutriflex LS) B. Braun Melsungen AG, Germany. The detailed characteristics of the PN admixtures are presented in Table 4.

Each PN admixture was prepared according to the manufacturer’s instructions, and then vitamins and trace elements were added. The Cernevit (Baxter, Poland) and Tracutil were added to Olimel, Nutriflex LS, and Nutriflex OS. The Soluvit N (Fresenius Kabi AB, Sweden) was dissolved in Vitalipid N Adult (Fresenius Kabi AB, Sweden), and such vitamin emulsion was added to Kabiven and Smofkabiven together with Addamel N (Fresenius Kabi AB, Sweden).

Based on the summary medicinal product characteristics, the infusion time for MPM and PN admixtures was determined. The volume ratios of the MPM and PN admixture in the infusion line were obtained by dividing the drug infusion rate by the PN admixture infusion rate. The calculated MPM:PN volume ratios used in the study were 1:1; 2:1, and 4:1.

### 4.2. Physicochemical Stability Assessment

Physical stability studies of PN admixtures included a visual examination and determination of pH, osmolarity, MDD of lipid emulsion, and zeta potential. All measurements were performed in triplicate for PN admixtures and MPM–PN admixture samples and expressed as mean ± standard deviation.

According to the European Pharmacopoeia [23], all samples were visually assessed against a black-and-white contrast background by two observers for lack of visible particles and color changing. The evaluation of pH was performed using pH-meter Seven Compact pH/ion S220 (Mettler Toledo, Greifensee, Switzerland). The MDD of lipid emulsion and zeta potential of PN admixtures were measured using Zetasizer Nano ZS (Malvern Instruments, Malvern, UK) at 25 °C according to US Pharmacopoeia [20]. The osmolarity was measured at room temperature using an 800CL TridentMed^®^ osmometer (Trident Med s.c., Warsaw, Poland).

Samples for particle size and zeta potential measurement were prepared following the same procedure, where 1 mL of MPM–PN admixture samples was diluted 10 times with water for injection. Then, 1 mL of diluted sample was transferred to a measurement cell for particle size and zeta potential determination, using DLS and laser Doppler electrophoresis (LDE) methods, respectively.

### 4.3. Acceptance Limits

To consider PN admixtures as compatible with MPM, the following criteria must be met: practically free from visible particles, no turbidity or precipitation may be detected by any of observers upon visual inspection, changes in pH after four hours of storage (ΔpH) <0.2, changes in osmolarity after four hours of storage (ΔOSM) < 5%, changes in zeta potential after four hours of storage (Δξ) <3 mV, the size of lipid droplets expressed as intensity-weighted MDD can not exceed the pharmacopeial limit of 500 nm, and no second fraction (dF2) of lipid droplets >1000 nm.

### 4.4. Statistical Analysis

The data were analyzed using Statistica 12 software (StatSoft, Cracow, Poland). Two-way analysis of variance (ANOVA) was used to determine the statistical significance occurring among the samples. The a priori level of significance was *p* < 0.05. In the case of a major effect or interaction, significant differences between the samples in t = 0 h and samples in t = 4 h were identified using Tukey’s honest significant difference (HSD) post hoc tests.

## 5. Conclusions

The assumed acceptance criteria for MPM compatibility with PN admixtures were met only for Nutriflex LS and Nutriflex OS in 1:1, 2:1, and 4:1 volume ratios. At the same time, the obtained results do not allow recommending coadministration of MPM via Y-site with Kabiven, Olimel, and Smofkabiven due to osmolarity fluctuations (MPM–Olimel), significant differences in zeta potential (MPM–Olimel, MPM–Smofkabiven), and the presence of the second fraction of particles >1000 nm (MPM–Kabiven, MPM–Olimel, and MPM–Smofkabiven).

## Figures and Tables

**Table 1:** Drug–parenteral nutrition admixtures interaction studies.

Compatibility Studies	Conclusions	References
Compatibility studies of Nutriflex Lipid Special and amoxicillin/clavulanic acid, calcium chloride, cefepime, cyclosporine, esomeprazole, fentanyl, fluorouracil, furosemide, magnesium sulfate, meropenem, metoclopramide, metronidazole, midazolam, morphine sulfate, noradrenaline, octreotide, ondansetron, pantoprazole, paracetamol, piperacillin/tazobactam, potassium phosphate, tacrolimus, tropisetron, vancomycin	Albumin, esomeprazole, pantoprazole, tropisetron, and 5-fluorouracil were not compatible with Nutriflex Lipid Special.	Bouchoud et al., 2013 [4]
RTU PN admixtures: Olimel N5E and Numeta G16E Drugs: ampicillin, ceftazidime, clindamycin, dexamethasone, fluconazole, fosphenytoin, furosemide, metronidazole, ondansetron, and paracetamol	Ampicillin, fosphenytoin, and furosemide precipitated when mixed with PN. Ceftazidime, clindamycin, dexamethasone, fluconazole, metronidazole, ondansetron and paracetamol were compatible.	Staven et al., 2017 [5]
Compatibility studies of ciprofloxacin and eighteen compounded PN admixtures for adults	Compatibility of ciprofloxacin with PN admixtures depended on drug concentration and calcium and magnesium molar ratio.	Gostyńska et al., 2019 [6]
Compatibility studies of vancomycin and five PN admixtures: Kabiven, Nutriflex Lipid Special, Olimel N9E, Nutriflex Omega Special, and Smofkabiven	Vancomycin was compatible with Kabiven, Nutriflex Lipid Special, and Nutriflex Omega Special.	Stawny et al., 2020 [7]
PN and lipid solutions used in a tertiary neonatal unit included a Starter, Standard Preterm and low carbohydrate PN, and SMOFLipid 20% with Vitalipid N infant and Soluvit N Drug: ibuprofen lysine	Ibuprofen lysine was compatible with tested PN admixtures and lipids.	Garcia et al., 2018 [8]
Compatibility and stability studies of linezolid with six compounded PN admixtures for adults	Linezolid was compatible and stable with tested PN admixtures.	Tomczak et al., 2019 [9]
Compatibility and stability studies of levetiracetam with two compounded PN admixtures for adults	Levetiracetam was compatible and stable with tested PN admixtures.	Aeberhard et al., 2017 [10]
Stability studies of ampicillin with two compounded PN admixtures for adults containing Lipofundin MCT/LCT or Lipidem	Administration of ampicillin with TPN admixture at the tested dose is possible when used ex tempore and with light protection.	Stawny et al., 2019 [11]
Compatibility studies of amiodarone with two compounded PN admixtures for adults containing Lipofundin MCT/LCT or Smofilipid	Amiodarone was physicochemically compatible with tested PN admixtures via a Y-site administration.	Mediavilla et al., 2019 [12]
Compatibility studies of pentoxifylline with six compounded PN admixtures used in neonatal intensive care	Pentoxifylline was physicochemically compatible with six PN admixtures used in neonatology.	Campbell et al., 2019 [13]
Compatibility studies of dexmedetomidine and three compounded PN admixtures for adults	Dexmedetomidine was compatible with tested PN admixtures.	Campos-Baeta et al., 2019 [14]
Compatibility studies of amiodarone, caffeine citrate, clindamycin, enalaprilat, epinephrine, fluconazole, fosphenytoin sodium, hydrocortisone, metoclopramide, midazolam, pentobarbital, phenobarbital, and rifampin with neonatal PN admixtures	Caffeine citrate, clindamycin, enalaprilat, epinephrine, fluconazole, fosphenytoin sodium, hydrocortisone, metoclopramide, and midazolam were compatible with tested PN admixtures. Amiodarone, pentobarbital, phenobarbital, and rifampin were not compatible with the neonatal TPN solution and should not be coadministered via Y-site injection.	Fox et al., 2013 [15]

**Table 2:** Characteristics of studied parenteral nutrition admixtures containing MPM.

Sample	MPM:PN Ratio	pH ± SD		Osmolality ± SD		Zeta Potential ± SD	
				(mOsm/kg H_2_O)		(mV)	
		0 h	4 h *	0 h	4 h	0 h	4 h
**MPM–Kabiven**	1:1	6.70 ± 0.00	6.66 ± 0.00	672 ± 1	685 ± 1	−19.0 ± 0.8	−19.2 ± 0.7
	2:1	7.07 ± 0.01	7.06 ± 0.01	528 ± 3	541 ± 4	−25.3 ± 1.1	−23.0 ± 0.8
	4:1	7.37 ± 0.01	7.32 ± 0.00	440 ± 3	437 ± 4	−25.6 ± 0.3	−25.9 ± 0.3
**MPM–Nutriflex LS**	1:1	6.47 ± 0.00	6.43 ± 0.00	977 ± 5	947 ± 4 **	−26.8 ± 1.6	−27.0 ± 1.2
	2:1	6.89 ± 0.00	6.86 ± 0.00	710 ± 3	725 ± 3 **	−32.8 ± 1.1	−32.2 ± 0.3
	4:1	7.20 ± 0.00	7.17 ± 0.01	546 ± 4	542 ± 5	−34.9 ± 1.1	−32.5 ± 0.7
**MPM–Olimel**	1:1	6.98 ± 0.00	6.85 ± 0.01	805 ± 11	892 ± 5 **	−20.6 ± 1.2	−32.1 ± 1.6 **
	2:1	7.22 ± 0.00	7.19 ± 0.00	630 ± 6	673 ± 4 **	−20.1 ± 1.4	−31.8 ± 5.9 **
	4:1	7.42 ± 0.01	7.35 ± 0.01	476 ± 1	516 ± 4 **	−20.4 ± 0.6	−32.0 ± 3.6 **
**MPM–Nutriflex OS**	1:1	6.44 ± 0.01	6.37 ± 0.00	957 ± 8	986 ± 1 **	−14.8 ± 0.4	−14.0 ± 0.3
	2:1	6.85 ± 0.00	6.8 ± 0.00	731 ± 1	749 ± 1 **	−19.2 ± 0.8	−22.0 ± 0.2
	4:1	7.15 ± 0.01	7.12 ± 0.01	549 ± 0	552 ± 0	−14.2 ± 0.9	−12.9 ± 0.2
**MPM–Smofkabiven**	1:1	6.41 ± 0.00	6.37 ± 0.01	901 ± 3	902 ± 2	−21.2 ± 0.6	−16.3 ± 0.4 **
	2:1	6.87 ± 0.01	6.85 ± 0.01	688 ± 4	675 ± 3 **	−23.5 ± 0.9	−20.5 ± 1.1
	4:1	7.23 ± 0.01	7.16 ± 0.01	514 ± 3	505 ± 3	−24.1 ± 1.4	−20.7 ± 0.8

SD—standard deviation; *—all pH results obtained after 4 h of storage were significantly different from t = 0 h values (*p* < 0.05); **—values obtained after 4 h of storage were significantly different from t = 0 h values (*p* < 0.05).

**Table 3:** Lipid droplet characteristics of studied parenteral nutrition admixtures containing meropenem (MPM).

Sample	MPM:PN Ratio	PDI ± SD	MDD ± SD		dF1 ± SD		dF2 ± SD	
			(nm)					
		4 h	0 h	4 h	0 h	4 h	0 h	4 h
**MPM–Kabiven**	1:1	0.05 ± 0.02	280.2 ± 3.2	280.2 ± 7.2	319.6 ± 4.2	319.6 ± 6.5	n.d.	n.d.
	2:1	0.17 ± 0.01	275.9 ± 4.5	275.9 ± 7.0	320.8 ± 3.7	320.7 ± 4.3	n.d.	4687 ± 7
	4:1	0.18 ± 0.01	277.8 ± 6.2	277.8 ± 6.3	326.9 ± 4.2	326.9 ± 6.2	n.d.	4972 ± 5
**MPM–Nutriflex LS**	1:1	0.06 ± 0.03	212.4 ± 2.2	212.4 ± 2.3	228.6 ± 2.3	228.6 ± 2.9	n.d.	n.d.
	2:1	0.07 ± 0.02	211.1 ± 1.9	208.3 ± 2.5	228.2 ± 2.1	227.8 ± 3.3	n.d.	n.d.
	4:1	0.05 ± 0.02	208.7 ± 2.5	208.7 ± 2.2	221.9 ± 3.4	221.9 ± 3.7	n.d.	n.d.
**MPM–Olimel**	1:1	0.16 ± 0.04	256.6 ± 1.4	266.8 ± 7.6 **	281.9 ± 5.8	296.2 ± 5.4	n.d.	5064 ± 9
	2:1	0.18 ± 0.01	254.4 ± 2.8	266.1 ± 4.6	281.4 ± 2.0	296.2 ± 4.7	n.d.	5190 ± 8
	4:1	0.21 ± 0.01	253.0 ± 2.6	262.8 ± 7.2	283.2 ± 8.1	282.4 ± 6.2	5170 ± 9	5389 ± 12
**MPM–Nutriflex OS**	1:1	0.07 ± 0.01	223.8 ± 1.7	220.9 ± 2.5	247.2 ± 1.3	238.4 ± 6.1	n.d.	n.d.
	2:1	0.08 ± 0.02	215.1 ± 1.3	216.4 ± 2.4	234.6 ± 4.3	238.2 ± 7.3	n.d.	n.d.
	4:1	0.06 ± 0.03	218.6 ± 1.2	219.5 ± 3.9	236.2 ± 7.6	239.7 ± 4.5	n.d.	n.d.
**MPM–Smofkabiven**	1:1	0.06 ± 0.01	237.1 ± 2.4	236.4 ± 2.1	272.8 ± 7.2	263.2 ± 3.0	n.d.	n.d.
	2:1	0.15 ± 0.01	234.5 ± 1.3	233.9 ± 0.4	258.1 ± 3.7	257.4 ± 4.6	n.d.	4996 ± 8
	4:1	0.16 ±0.01	234.0 ± 2.5	232.0 ± 0.2	265.4 ± 3.5	255.0 ± 4.3	n.d.	5151 ± 10

SD—standard deviation; dF1—first fraction of lipid emulsion particles; dF2—second fraction of lipid emulsion particles n.d.—not detected; **—values obtained after 4 h of storage were significantly different from t = 0 h values (*p* < 0.05).

**Table 4:** Composition of tested parenteral nutrition admixtures.

	Kabiven	Nutriflex LS	Olimel	Nutriflex OS	SmofKabiven
	**g/1000 mL**				
**Alanine**	4.7	6.8	8.3	6.8	7.1
**Arginine**	3.3	3.8	5.6	3.8	6.1
**Aspartic acid**	1.0	2.1	1.7	2.1	-
**Glutamic acid**	1.6	4.9	2.9	4.9	-
**Glicyne**	2.3	2.3	3.9	2.3	5.6
**Histidine**	2.0	1.8	3.4	1.8	1.5
**Izoleucine**	1.6	3.3	2.9	3.3	2.6
**Leucine**	2.3	4.4	3.9	4.4	3.8
**Lisyne**	2.7	3.2	4.5	3.2	3.4
**Methionine**	1.6	2.7	2.9	2.7	2.2
**Phenylalanine**	2.3	4.9	3.9	4.9	2.6
**Proline**	2.0	4.7	3.4	4.7	5.7
**Serine**	1.3	4.2	2.3	4.2	3.3
**Taurine**	-	-	-	-	0.54
**Threonine**	1.6	2.6	2.9	2.6	2.2
**Tryptophan**	0.6	0.8	0.9	0.8	1.0
**Tyrosine**	0.1	-	0.1		0.2
**Valine**	2.1	3.6	3.7	3.6	3.1
**Total amino acids**	33.1	56.1	56.9	56.1	50.8
**Nitrogen**	5.3	8.0	9.0	8.0	8.1
**Glucose**	97.4	144.0	110.0	144.0	126.6
**LCT**	39.0	20.0	8.0	20.0	11.4
**MCT**	-	20.0	-	16.0	11.4
**Olive oil**	-	-	32.0	-	9.5
**Omega-3 acids**	-	-	-	4.0	5.7
**Total lipids**	39.0	40.0	40.0	40.0	38.1
	**mmol/1000 mL**				
**Sodium**	31.2	53.6	35.0	53.6	40.6
**Potassium**	23.4	37.6	30.0	37.6	30.5
**Magnesium**	3.9	4.2	4.0	4.2	5.1
**Calcium**	1.9	4.2	3.5	4.2	2.6
**Zinc**	-	0.032	-	0.032	0.041
**Chlorides**	45.5	48.0	45.3	48.0	35.2
**Phosphates**	37.7	16.0	15.0	16.0	12.9
**Acetates**	9.7	48.0	53.3	48.0	106.3

## Data Availability

Data is contained within the article.
